# Determinants of immunosuppressive therapy in renal transplant recipients: an Italian observational study (the CESIT project)

**DOI:** 10.1186/s12882-023-03325-9

**Published:** 2023-10-27

**Authors:** Alessandro C. Rosa, Marco Finocchietti, Nera Agabiti, Paolo Menè, Maria Elena Bracaccia, Arianna Bellini, Marco Massari, Stefania Spila Alegiani, Lucia Masiero, Gaia Bedeschi, Massimo Cardillo, Ersilia Lucenteforte, Giuseppe Piccolo, Olivia Leoni, Eliana Ferroni, Silvia Pierobon, Maurizio Nordio, Stefano Ledda, Donatella Garau, Marina Davoli, Antonio Addis, Valeria Belleudi, on behalf of CESIT study group, Francesca R. Poggi, Maria Lucia Marino, Claudia Marino, Ursula Kirchmayer, Andrea Ricci, Francesca Puoti, Vito Sparacino, Pamela Fiaschetti, Silvia Trapani, Alessandra Oliveti, Daniela Peritore, Lorella Lombardozzi, Manuel Zorzi, Martina Zanforlini, Arianna Mazzone, Michele Ercolanoni, Andrea Angelo Nisic, Paolo Carta, Valentina Ientile, Luca L’Abbate, Matilde Tanaglia, Gianluca Trifirò, Ugo Moretti

**Affiliations:** 1 Department of Epidemiology, Lazio Regional Health Service, ASL Roma 1, Via Cristoforo Colombo, 112, 00147 Rome, Italy; 2 Department of Clinical Sciences, Division of Nephrology, University of Rome La Sapienza, Sant’Andrea University Hospital, Rome, Italy; 3 National Centre for Drug Research and Evaluation, Istituto Superiore Di Sanità, Rome, Italy; 4 Italian National Transplant Centre, Istituto Superiore Di Sanità, Rome, Italy; 5 Department of Clinical and Experimental Medicine, University of Pisa, Pisa, Italy; 6 Regional Transplant Coordination, Lombardy Region, Milan, Italy; 7 Department of Health of Lombardy Region, Epidemiology Observatory, Milan, Italy; 8 Azienda Zero of the Veneto Region, Padua, Italy; 9 General Directorate for Health, Sardinia Region, Cagliari, Italy

**Keywords:** Immunosuppressive therapy, Determinants, Kidney, Real world evidence, Spatio-temporal variability

## Abstract

**Background:**

Very scanty evidence is available on factors influencing the choice of immunosuppressive drug therapy after kidney transplantation.

**Methods:**

An Italian multiregional real-world study was conducted integrating national transplant information system and claims data. All patients undergoing kidney transplantation for the first time during 2009–2019 (incident patients) were considered. Multilevel logistic models were used to estimate Odds Ratio (OR) and corresponding 95% Confidence intervals. Factors with statistically significance were identified as characteristics associated with treatment regimens: cyclosporin-CsA vs tacrolimus-Tac and, within the latter group, mTOR inhibitors vs mycophenolate-MMF.

**Results:**

We identified 3,622 kidney patients undergoing transplantation in 17 hospitals located in 4 Italian regions, 78.3% was treated with TAC-based therapy, of which 78% and 22% in combination with MMF and mTOR, respectively. For both comparison groups, the choice of immunosuppressive regimens was mostly guided by standard hospital practices. Only few recipient and donor characteristics were found associated with specific regimen (donor/receipt age, immunological risk and diabetes).

**Conclusions:**

The choice of post-renal transplant immunosuppressive therapy seems to be mostly driven by standard Centre practices, while only partially based on patient’s characteristics and recognized international guidelines.

**Supplementary Information:**

The online version contains supplementary material available at 10.1186/s12882-023-03325-9.

## Introduction

During the past two decades, the introduction of new immunosuppressive drugs has represented the key to the success of solid organ transplantation. Several immunosuppressive agents block specific mechanisms underlying the adaptive response. Standard immunosuppression protocol for maintenance phase consists of calcineurin inhibitors “CNI” (Cyclosporine “CsA” or Tacrolimus “Tac”) with antimetabolite (Mycophenolate “MMF” or Azathioprine “Aza”) or mTORi (Everolimus “EVE” or Sirolimus “SIR”) plus corticosteroids, mainly Prednisone “PRED” [1, 2]. Although in several countries the most frequent triple-drug regimen included Tac, MMF and PRED [3], in Europe data on drug treatment in early post-transplant phase as well as data on the variation per year and per region are limited.

A recent article shows a high heterogeneity of the immunosuppressive drug combinations employed in the maintenance phase of solid organ transplantation in the Italian context [4].

The possible reasons for choosing a certain regimen of immunosuppressive therapy over other schemes in terms of type or number of active agents prescribed, are poorly investigated. The choice of therapy should take into account both the efficacy to prevent graft rejection and potential long-term side effects, so that demographic and clinical characteristics of donor and graft recipient should drive the choice of treatment [5, 6].

Furthermore, the choice of immunosuppressive regimen may vary across hospitals; instead, so a thorough evaluation of patients’ characteristics should drive the immunosuppressant regimen on the basis of a multilevel approach [7].

The aim of our study was to assess recipient, donor and transplant characteristics associated with immunosuppressive maintenance strategy in a kidney transplant cohort across four Italian districts (regions), taking into account the local hospital’s policies.

## Methods

### Study design

A retrospective observational study on determinants related to drug utilization patterns in a cohort of kidney transplant patients, resident in 4 Italian regions (Lombardy, Veneto, Lazio, Sardinia) was performed. The transplant patients’ cohort was identified through data available in regional administrative healthcare databases linked to national transplant information system (SIT) using a common data model and an open-source tool for distributed analyses, “*The Shiniss*”. The study design, health information flows used, the institutional framework, record-linkage procedures are described in more detail elsewhere [8].

**Fig. 1:**
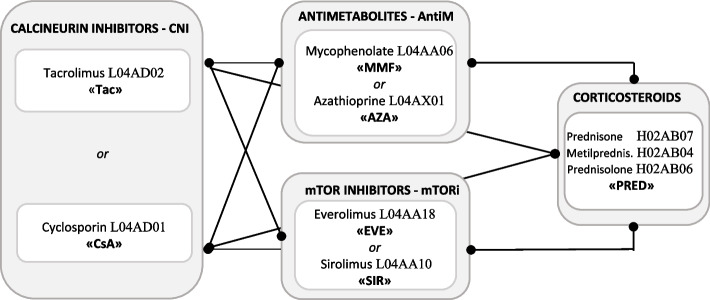
Therapeutic regimens in the maintenance phase. Note: In the grey box are the therapeutic classes of immunosuppressive drugs, in clear boxes the actual agents prescribed. Links represent possible concomitant use of drugs

Briefly, the study included all patients who underwent incident kidney transplant in the years 2009–2019, surviving 30 days after discharge, with at least one immunosuppressive dispensation in the maintenance phase after hospital discharge. Patients with previous immunosuppressive treatment were excluded. The complexity of the treatment regimens is schematized in the Fig. 1.

Based on the dispensation retrieved during the 30 days after transplant discharge (*index therapy*), patients treated with CNI therapies were identified. More specifically, therapeutic regimen groups were classified in CsA- or Tac-based therapies, considered alone or in combination with other active agents, i.e. MMF or mTORi with/without PRED. With respect to the use of antimetabolite drugs together with Tac, the focus in this paper is mainly on the comparison with MMF since, in the renal setting, the frequency of combination of Azathioprine (AZA) with Tac is low [2].

Patterns of use of immunosuppressive drugs have been described in details in a previous article [4]. In this paper, the comparison groups in study were: CsA vs Tac (1^st^ comparison group) and Tac + mTORi vs Tac + MMF (2^nd^ comparison group).

### Recipient, donor and transplant characteristics

In addition to the contextual variables like hospital where the transplant has been performed, year of discharge, length of stay during index hospitalization, the following covariates related to recipient were tested as factors potentially related to immunosuppressive therapy: sex, age, cardio-cerebrovascular disease, respiratory diseases, tumors, thyroid gland disorders, diabetes, lipid metabolism disorders and obesity, hematologic diseases, hypertension. From SIT, we tested variables of clinical interest: transplant indications, type of dialysis, the distance between first dialysis and transplantation. Moreover, panel reactivity antibodies (PRA), a test to detect preformed recipient antibodies through the use of a panel of typifying cells and the number of mismatches, a target categorical variable denoting the level of compatibility between donor and recipient.

With respect to donor characteristics, we explored sex, age, type of donor (deceased/living), previous infections and malignancies.

In detail, data sources, time windows and criteria used to define covariates can be found in a previous article [8].

### Statistical analysis

Maintenance immunosuppressive treatment regimens were plotted using *sunburst charts*. We described the most frequent combinations of immunosuppressive drugs administered to patients and underlined the two comparison groups of interest (CsA vs Tac; mTORi vs MMF Tac–based). Treatment groups represented the dependent variable in the analyses.

Clinical, demographic and contextual variables were presented separately between the two comparison subgroups as column-wise frequencies and percentages for categorical variables and mean/median for continuous variables. Only variables with a threshold frequency above 5% in the overall cohort were considered.

In order to evaluate the role of transplant centers in the choice of drug therapy, the proportion of patients treated with the various immunosuppressive combinations was stratified by transplant center.

Considered the hierarchical data structure, patients are nested within hospitals, a logistic multilevel model was performed in order to account for potential intra-class correlation and to consider the clustered nature of the data. Moreover, our goal was to investigate sources of variations within- and between-clusters, to describe variables predicting individual differences and to describe which variables predict cluster-level differences [9]. The variance components were expressed in terms of Median Odds Ratio (MOR), a measure that quantifies the variability in receiving CsA versus Tac for the 1^st^ comparison and Tac + mTORi versus Tac + MMF in the 2^nd^ comparison between clusters, in this case between different hospitals of discharge [10]. The MOR quantifies the variation in dependent variable between clusters by comparing two persons from two randomly chosen different hospitals. For instance, consider two patients with the same covariates, chosen randomly from two different clusters. The MOR is the median odds ratio between the patient of higher propensity and one of lower propensity [11]. This measure is always equal or greater than 1.00.

A MOR equal to 1.00 indicates no variability between clusters; as the variability between group increases, the value of the measure increases.

In the first step, in order to quantify the crude variability of treatment choices among hospitals performing transplants, the MOR was estimated using an intercept-only model. In a second step, the MOR was estimated controlling for patient characteristics, in order to ensure that different composition of patients within groups (in terms of age, sex, comorbidities) did not influence estimates of variance.

Age and sex of receiving patient were considered as a priori determinants of the drug therapy choice, others factors were selected by stepwise logistic regression methods. For both comparisons, a multilevel model (OR; 95%CI) was applied to identify determinants of index therapy, after correction for variability between hospitals.

The model's ability to predict treatment patterns by taking into account, separately and together, hospital and individual patient characteristics was evaluated through the Receiver Operating Characteristic (ROC) curve [12]. In particular, for both groups of comparison, the AUC (Area Under the ROC Curve) was estimated; the latter measures the entire two-dimensional area under the entire ROC curve, providing an aggregate measure of performance across all possible classification thresholds.

Data management and statistical analyses were carried out using Sas software (Sas Enterprise Guide Vers 7.15, SAS Institute Inc., Cary, NC, USA).

## Results

After applying exclusion criteria and identifying index therapy administered to patients (Figure S1), the cohort of kidney transplant recipients in the receiving CNI treatment was limited to 3,622 subjects (78.3% treated with Tac).

**Fig. 2:**
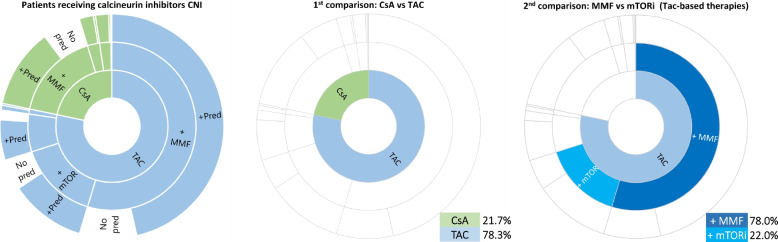
Immunosoppressive treatment patterns and comparison groups among kidney transplant patients. Note: The comparison groups are highlighted with more intense colouring

Among patients receiving Tac-based index therapy, 559 (22.0%) were treated in combination with mTORi. Through sunbursts graphs (Fig. 2), it is possible to visualize the proportion of patients treated with specific therapies and comparison groups.

**Fig. 3:**
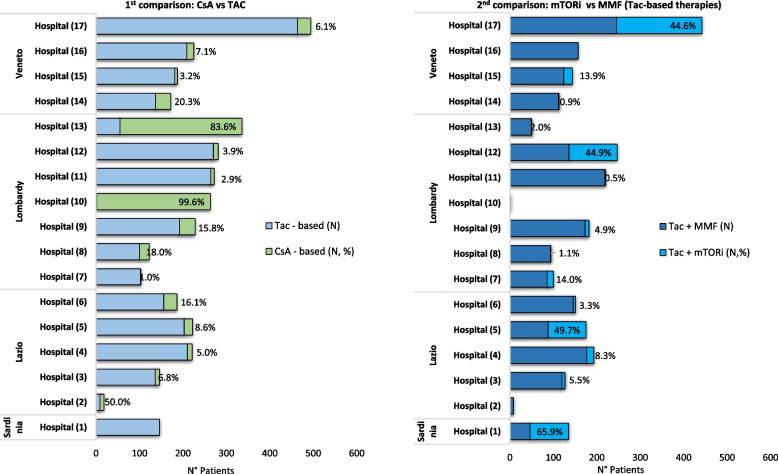
Heterogeneity of treatment patterns between transplant centers

Analysis of therapy at the time of discharge from the hospital reveals a strong heterogeneity of index therapy protocols applied to patients belonging to the 1^st^ comparison group (CsA vs Tac) and, among patients treated with Tac, belonging to the 2^nd^ comparison (mTORi vs MMF) (Fig. 3).

Comparing CsA vs Tac, the proportion of patients treated with CsA in the cohort ranges from 0% to 99.6%, with a further regional gradient easily discernible in the graph. In support of that, the MOR calculated taking into account only the intercept is 10.2 (*p*-value 0.005). Comparing mTORi vs MMF, the proportion of patients treated with mTORi in the Tac-based subpopulation varies from 0% to 65.9%. The crude MOR is 6.78 (*p*-value 0.008).

Variability of treatment among the prescribing transplant centers is less striking excluding confounding effects of 2 Centers (Lombardy #10 and #13, who rely on CsA for 99.6% and 83.6% of their patients) (Fig. 3). The same concept could be applied to the choice of mTORi, which are prescribed in combination with Tac by 4 Hospitals (Sardinia #1, Lazio #5, Lombardy #12, and Veneto #17), ranging from 44.6 to 65.9% of patients as an alternative to the more widely used MMF.

Characteristics by comparison groups are shown in Table 1.

**Table 1:**
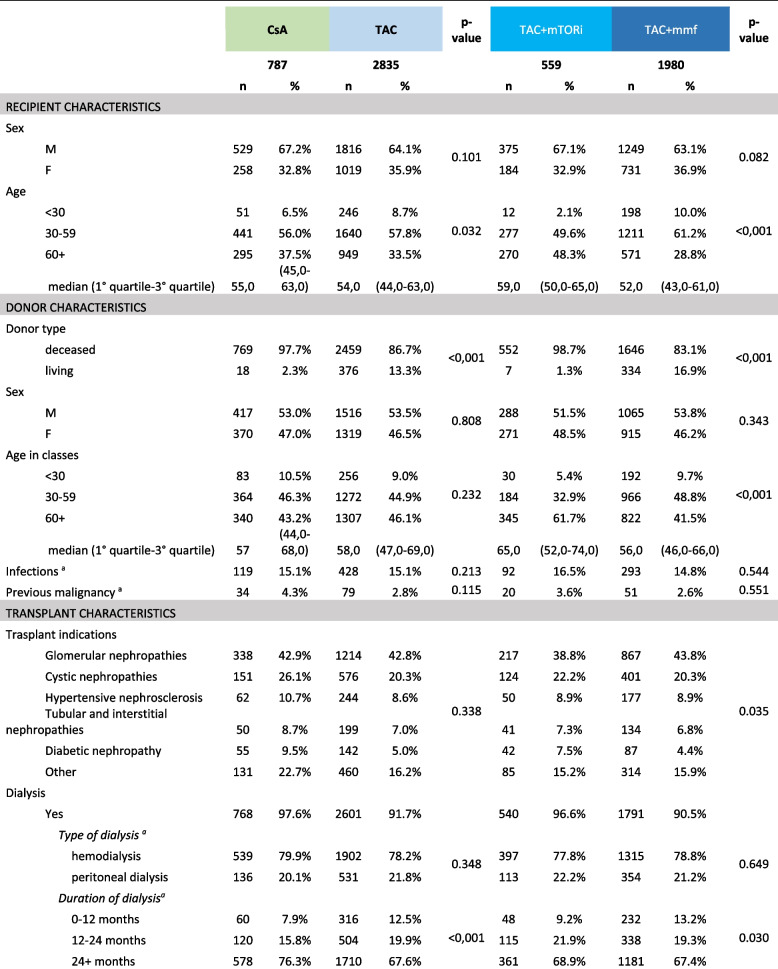

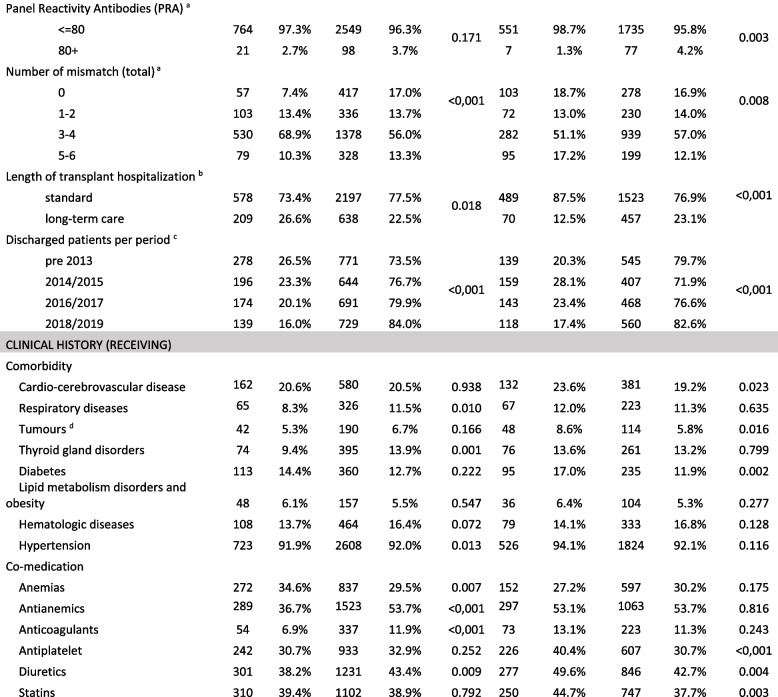
Recipient, donor and transplant characteristics

^a^the percentages given in the table do not take into account possible missing observations

^b^the definition of "Long term care" considers observations beyond the 75^th^ percentile (20 days) of the distribution

^c^Row percentages. Data partially available for years prior to 2013

^d^Tumours of the kidney are not considered considered

The median age of recipients was similar between CsA (55 years) and TAC users (54 years); the proportion of living donors appears to be significantly higher for patients starting therapy on CsA (13.3%) than for those treated with Tac (2.3%). In relation to clinical information retrieved from SIT, among dialysis-treated patients, CsA-treated patients are more likely to be associated with a dialysis course longer than 24 months (CsA 76.3% vs Tac 67.6%). The variable 'number of mismatches' shows a different distribution of coding classes, particularly in the '0' class (CsA 7.4% vs Tac 17.0%) and the '3–4' class (CsA 68.9% vs Tac 56.0%).

Concerning patients treated with CsA, there is a higher proportion of inpatients treated over a prolonged period of time during the index hospitalization (CsA 26.6% vs Tac 22.5%). It can be seen that over time, the proportion of patients with index therapy in Tac increased (pre 2013, it was 71.7%, reaching 84.0% in 2018–2019).

Furthermore, patients treated with Tac show a higher prevalence of comorbidities such as respiratory diseases (CsA 8.3% vs Tac 11.5%) and thyroid gland disorders (CsA 9.4% vs Tac 13.9%); there is a different concomitant use for some drug categories, such as anti-anaemic drugs (CsA 36.7% vs Tac 53.7%).

In the 2^nd^ comparison group (Tac-based therapies with addition of other immunosuppressors), a higher median age of the recipient patient and donor is shown in mTOR user (59 vs 52 and 65 vs 56 years, respectively). The proportion of living donors appears to be significantly different (mTORi 1.3% vs MMF 16.9%).

The proportion of patients treated with high PRA (80 +) was higher among patients treated with mTORi (4.2% vs 1.3%). The variable 'number of mismatches' showed a different distribution of coding classes, particularly in the '3–4' class (mTORi 51.1% vs MMF 57.0%).

With respect to the patient's clinical history, a different proportion of patients with cardio-cerebrovascular disease (mTORi 23.6% vs MMF 19.2%) and diabetes (mTORi 17.0% vs MMF 11.9%) emerged. With regard to additional drugs used, we observed different proportions of patients treated with antiplatelet (mTORi 40.4% vs MMF 30.7%) and statins (mTORi 44.7% vs MMF 30.7%).

**Fig. 4:**
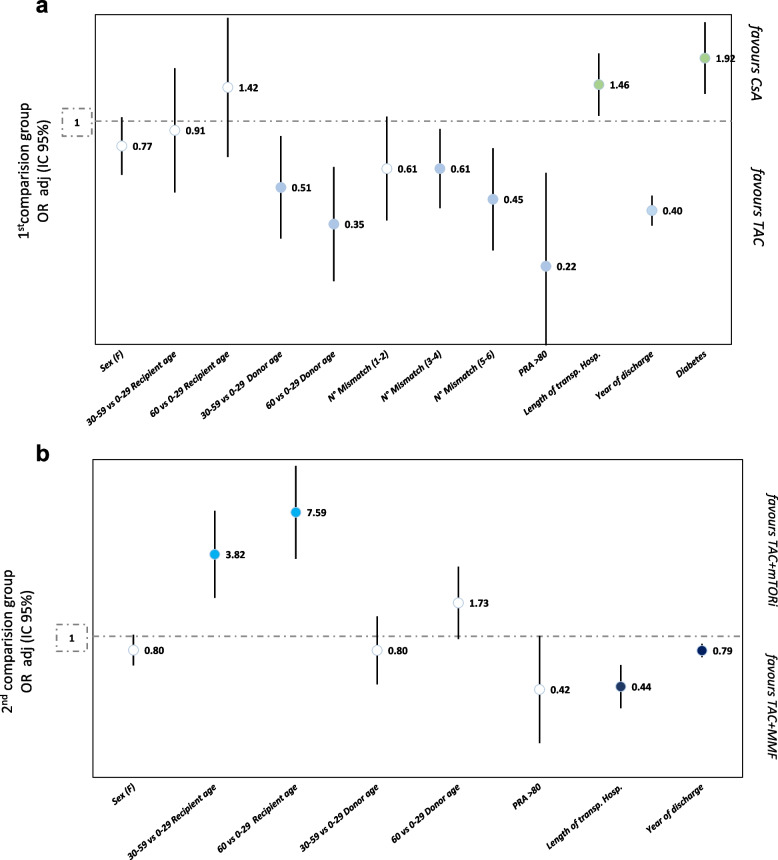
**a** Determinants related to index therapy: CsA vs TAC. **b** Determinants related to index therapy: Tac + mTORi vs TAC + MMF

The multivariable models show factors associated with maintenance immunosuppressive therapies, net of explained variability by transplant centers (Fig. 4a, b).

Within the 1^st^ comparison group (CsA vs Tac), the effect of age of donor was clear with respect to the reference category (age less than 30 years): prescription of Tac increased in the age group’30–59’ years (OR: 0.51; 95% CI: 0.30–0.86) and further in the group’60 + ’ years (OR: 0.35; 95% CI: 0.19–0.62). Increasing the “number of mismatches” is a determinant of immunosuppressive therapy with Tac (3–4 Mismatch OR: 0.61; 95% CI: 0.41–0.92, 5–6 Mismatch OR: 0.45; 95% CI: 0.26–0.76), like having “PRA” + 80 (OR: 0.22; 95% CI: 0.22–0.59). A contextual factor determining CsA-based index therapy is the length of the index hospitalization in which the transplant is performed (OR: 1.49; 95% CI: 1.08–2.06). More recent years are increasingly related with a greater use of Tac (OR: 0.44; 95% CI: 0.38–0.50). In the supplementary materials, an area graph makes it possible to visualise the variation over time of the proportion of patients treated with the immunosuppressors of interest (Figure S2).

About comorbidities, diabetes is factor related with index CsA-based therapy (OR: 1.90; 95% CI: 1.31–2.77). After controlling for patients’ characteristics, we detected variation between hospitals (MOR: 14.56 *p*-value: 0.004). The applied model considering both facility and patient characteristics, achieve a high level of patient classification to therapy (AUC = 0.94) (Figure S3a).

Within the 2^nd^ comparison group, bordering on statistical significance female patients are more related of being treated with MMF (OR: 0.80; 95% CI: 0.62–1.03). The effect of age of receiving was clear, with respect to the reference category (age less than 30 years): being treated with mTORi increased in the age group’30–59’ years (OR: 3.82; 95% CI: 1.88–7.78) and further in the group’60 + ’ years (OR: 7.59; 95% CI: 3.56–16.21), despite reducing the accuracy of the estimate. Contextual factors such as prolonged-hospitalization after transplant (OR: 0.44; 95% CI: 0.31–0.63) and recent years (OR: 0.80; 95% CI: 0.71–0.86) are related with a greater use of MMF. After controlling for patients’ characteristics, we detected a clear variation among hospitals (MOR: 7.03 *p*-value: 0.008). The applied models, considering both hospital level and patient characteristics, achieve a satisfactory level of patient classification to therapy (AUC = 0.88) (Figure S3b).Given the changes in treatment patterns over time, we decided to replicate the main analysis in the subgroup of patients considering only the most recent years of observation, from 2014 onwards. The results are comparable with the models presented above (Table S1A-B).

## Discussion

This is the first paper, to our knowledge, that investigated the criteria underlying the choice of the immunosuppressive regimen prescribed immediately following a kidney transplant. We herein report on an important variability of treatment regimens among the transplant centers reviewed, both in patients receiving CNI inhibitor-based therapies and, among Tac users, those treated with mTORi or antimetabolites. Much of the observed heterogeneity depends on a few hospitals; this implies that it might be useful to study determinants within centers, but the low numbers do not allow accurate estimates. A more detailed knowledge of the internal protocols of transplant centers would be useful to better understand the real motivations behind the choice of treatment.

Net of the role of the discharging hospital, a few individual and transplant characteristics were found associated with specific regimens. In the first comparison, increasing donor age and incompatibility between donor and recipient were associated with Tac-based therapy; in contrast, a clinical history of diabetes in recipients was more likely associated with a CsA-based therapy. In terms of contextual factors, there has been an increased use of Tac-based therapies in recent years, while prolonged transplant’s hospitalization was related to CsA-based therapies.

Furthermore, as the patient's age increases, earlier protocols and standard length of hospitalizations were associated with a greater use of Tac combined with mTORi.

Our findings showed that treatment choice was mainly driven by hospital’s standard practices. The key role of the hospital in establishing the choice of regimen was already shown by Axelrod D et al. [7] in the US context; actually, in this study, only a few case-level factors, including immunological risk, were identified as being associated with therapy. Donor's age may have an adverse effect on graft function [13], an elevated number of HLA antigen mismatches and PRA > 80 indicate a high risk of graft failure and reject [14]; consequently, the association found in our analysis between these elements and TAC index therapy could be related to the greater efficacy of TAC in preventing rejection and graft failure compared to CsA [15]. Along this line, the increase in TAC therapy within discharge years is probably related to the body of evidence published over the last years that has concurred in identifying TAC as the calcineurin inhibitor of choice in post-transplant patients [16]. Finally, our analysis revealed the diabetogenic potential of TAC-based therapy; as a matter of fact, patients with diabetes were more frequently assigned to CsA therapy [17].

Regarding the second comparison, the association with high recipient’s age and the use of mTORi may be related to the antineoplastic effect of these drugs and the increase of cancer risk with age [18].

In the era of precision medicine, right treatments to the right patients at the right time, the role of the patient's clinical history in determining treatment choices should probably be held stronger. Individual characteristics emerged from our analysis suggest that in the years considered there was no real consensus on the most effective therapeutic strategy in kidney patients;

The guidelines [19] on maintenance immunosuppression for kidney recipients provide general indications on the best drug combination to use after transplantation, without considering subpopulations with specific characteristics or comorbidities, nor do they indicate possible changes of therapy or precautions to be taken with these patients. The great heterogeneity in the choice of drug treatment observed, suggests the need to improve evidence, by RCT or RWE, in the risk–benefit profile of immunosuppressive strategies in specific subgroup population, including patients with comorbidities, such as hypertension hyperparathyroidism, cardiovascular diseases, history of chronic infections, in order to better tailoring immunosuppressive strategy on patient’s characteristics [20].

### Strengths and limitations

This is a multi-regional study: the Regions included perform approximately 45% of transplants nationwide [8].

The use of specific graphical approaches allows the representation of the complexity of immunosuppressive drug therapies dispensed in clinical practice.

The observational nature of this study has certain limitations: while allowing the integration of many different data sources, many clinical parameters were not available (including information from biopsy of the recipient); moreover, misclassification of the index therapy could be due to a lack of available data on out-of-pocket drug purchase and on the drugs dispensed at the hospital level, during the post-transplant hospitalization.

Furthermore, reasons behind specific treatment protocols implemented by individual discharge hospitals cannot be quantified by current administrative flows.

Although this analysis covers four major Italian regions, among which the two most populated (÷ 16.000.000 inhabitants), it may not be fully representative of the actual clinical practice nationwide.

## Conclusion

Our results show that immunosuppressive regimens applied to kidney transplant patients in the immediate post-discharge period are fairly heterogeneous, seems to be mostly related to standard practices of centers in charge of the patient. However, some recipient and donor characteristics emerge from the analyses: further research could be useful to investigate the underlying reasons of it and whether other factors, not detectable through current data flows, may better explain this phenomenon.

### Supplementary Information

**Additional file 1: Figure S1.** Flow chart.

**Additional file 2: Figure S2.** Proportion of patients treated with immunosuppressive therapies over time.

**Additional file 3: Figure S3.** (a,b). ROC curves by comparison groups.

**Additional file 4: Table S1. **A-B. Determinants related to index therapy: CsA vs TAC, Tac+ mTORi vs TAC+MMF (selection period 2014-2019).

## Data Availability

The datasets generated and/or analyzed during the current study are not publicly available because of privacy reasons. For further information please contact the corresponding author: v.belleudi@deplazio.it.
