# Supplementary material for: Determinants of immunosuppressive therapy in renal transplant recipients: an Italian observational study (the CESIT project)
Source: BMC Nephrol. 2023 Oct 27;24:320. doi: 10.1186/s12882-023-03325-9 (PMC10604923; doi:10.1186/s12882-023-03325-9)
Supplement: Supplementary file 1 — Additional file 1: Figure S1. Flow chart. [file 12882_2023_3325_MOESM1_ESM.docx]

*Supplementary materials*

Figure S1. Flow chart.

Kidney Transplant Patients (TPs) in the study period (n=7,988)

with single transplant (n=7,988; 100.0%)

resident in the study region (n=5,318; 66.6%)

with incident transplant (n=4,596; 86.4%)

survived 30 days post-discharge, with at least one immunosuppressive prescription within 30 days post-discharge (n=4,335; 94.3%)

linked with transplant information systems (n=4,029; 92.9%)

**NO CNI**

**407 (10.1%)**

**TAC**

**2835 (70.4%)**

**CsA**

**787 (19.5%)**

**TAC+mTOR**

559 (19.7%)

**TAC+MMF**

1980 (69.8%)

**TAC mono**

261 (9.2%)

**TAC+other**

35 (1.2%)
