# Supplementary material for: Determinants of immunosuppressive therapy in renal transplant recipients: an Italian observational study (the CESIT project)
Source: BMC Nephrol. 2023 Oct 27;24:320. doi: 10.1186/s12882-023-03325-9 (PMC10604923; doi:10.1186/s12882-023-03325-9)
Supplement: Supplementary file 2 — Additional file 2: Figure S2. Proportion of patients treated with immunosuppressive therapies over time. [file 12882_2023_3325_MOESM2_ESM.pptx]

## Slide 1
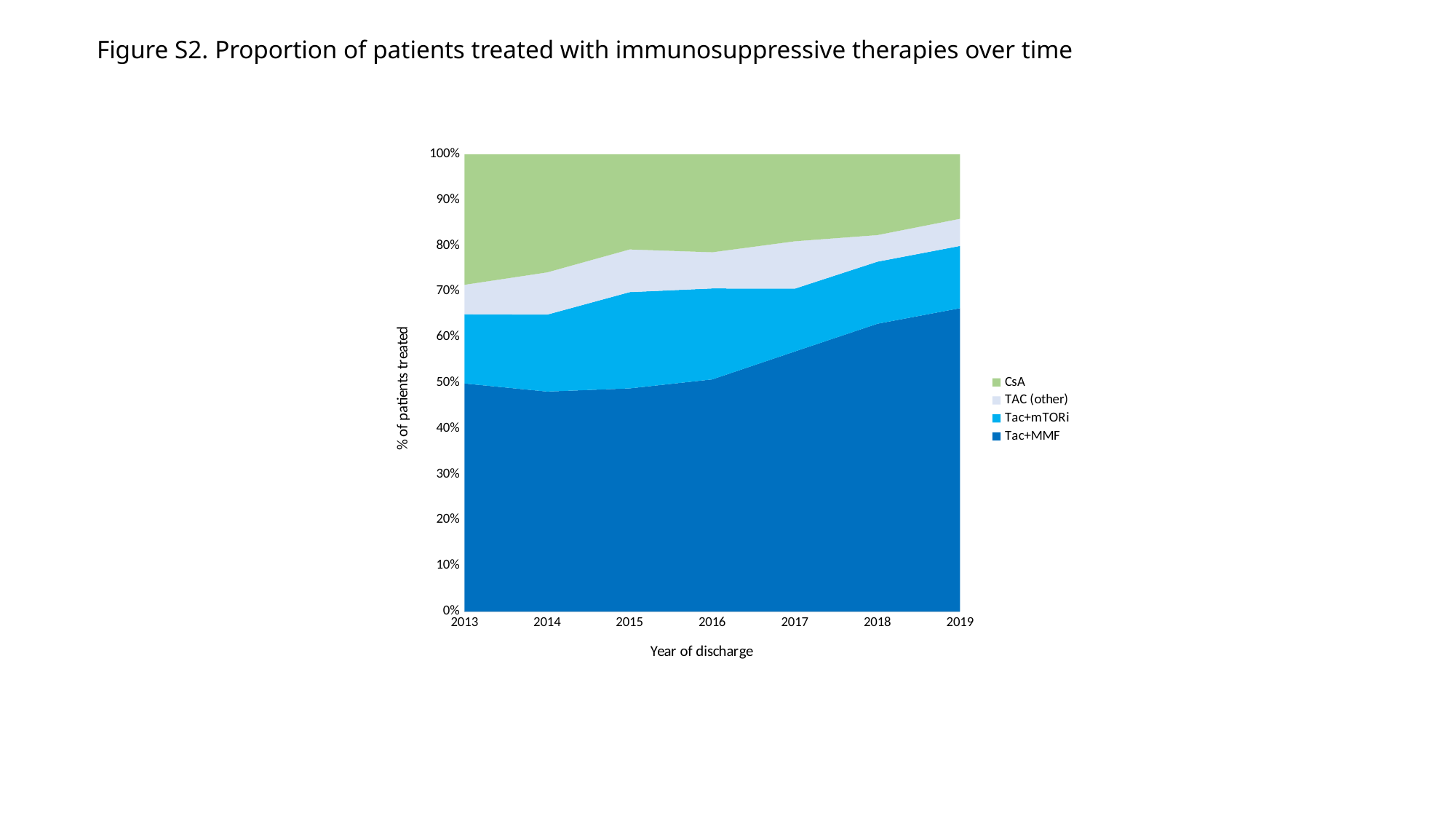

# Figure S2. Proportion of patients treated with immunosuppressive therapies over time
### Chart
| Category | Tac+MMF | Tac+mTORi | TAC (other) | CsA |
|---|---|---|---|---|
| 2013 | 201.0 | 61.0 | 26.0 | 115.0 |
| 2014 | 203.0 | 71.0 | 39.0 | 109.0 |
| 2015 | 204.0 | 88.0 | 39.0 | 87.0 |
| 2016 | 199.0 | 78.0 | 31.0 | 84.0 |
| 2017 | 269.0 | 65.0 | 49.0 | 90.0 |
| 2018 | 292.0 | 63.0 | 27.0 | 82.0 |
| 2019 | 268.0 | 55.0 | 24.0 | 57.0 |
