# Supplementary material for: Determinants of immunosuppressive therapy in renal transplant recipients: an Italian observational study (the CESIT project)
Source: BMC Nephrol. 2023 Oct 27;24:320. doi: 10.1186/s12882-023-03325-9 (PMC10604923; doi:10.1186/s12882-023-03325-9)
Supplement: Supplementary file 3 — Additional file 3: Figure S3. (a,b). ROC curves by comparison groups. [file 12882_2023_3325_MOESM3_ESM.pptx]

## Slide 1
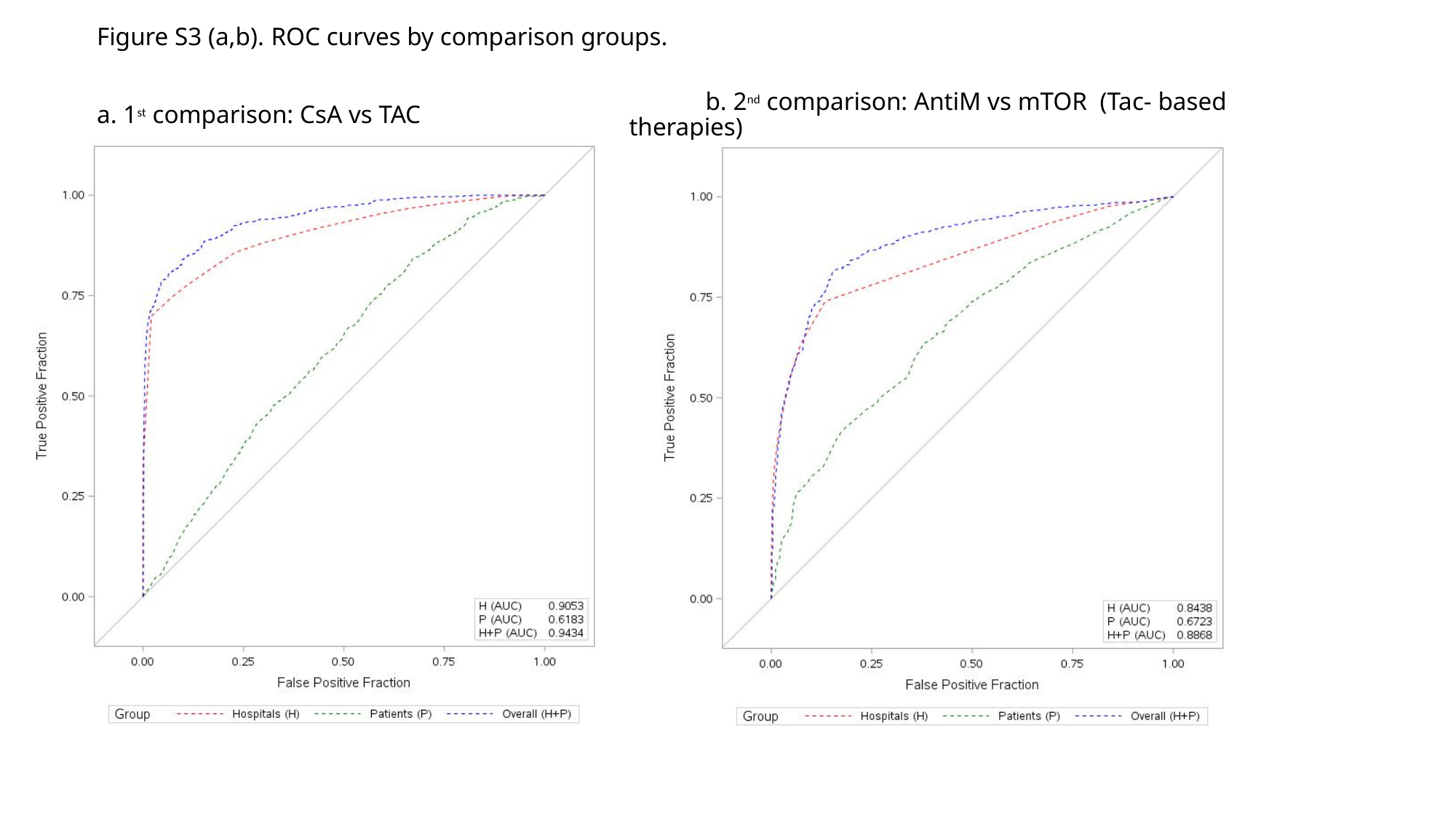

# Figure S3 (a,b). ROC curves by comparison groups.
a. 1st comparison: CsA vs TAC
 b. 2nd comparison: AntiM vs mTOR (Tac- based therapies)
