# Supplementary material for: Determinants of immunosuppressive therapy in renal transplant recipients: an Italian observational study (the CESIT project)
Source: BMC Nephrol. 2023 Oct 27;24:320. doi: 10.1186/s12882-023-03325-9 (PMC10604923; doi:10.1186/s12882-023-03325-9)
Supplement: Supplementary file 4 — Additional file 4: Table S1. A-B. Determinants related to index therapy: CsA vs TAC, Tac+ mTORi vs TAC+MMF (selection period 2014-2019). [file 12882_2023_3325_MOESM4_ESM.docx]

Table S1A-B. Determinants related to index therapy: CsA vs TAC, Tac+ mTORi vs TAC+MMF (selection period 2014-2019)

| **A) CsA vs TAC N=2288** | | | | |
| --- | --- | --- | --- | --- |
| **Variable** | **OR** | **95% CI** | | **p Value** |
| **Sex** |  |  |  |  |
| *F* | 0.72 | 0.45 | 1.13 | 0.1536 |
| *M* | 1.00 | *Reference Group* | | |
| **Recipient age** |  |  |  |  |
| 60+ | 0.57 | 0.19 | 1.77 | 0.0028 |
| *30-59* | 0.29 | 0.10 | 0.81 |  |
| *0-29* | 1.00 | *Reference Group* | | |
| **Donor age** |  |  |  |  |
| 60+ | 0.23 | 0.09 | 0.59 | 0.0088 |
| *30-59* | 0.30 | 0.13 | 0.73 |  |
| *0-29* | 1.00 | *Reference Group* | | |
| **Length of transplant hospitalization** | 1.74 | 1.07 | 2.83 | 0.0264 |
| **Year of discharge** | 0.37 | 0.27 | 0.50 | <.0001 |
| **Diabetes** | 2.80 | 1.63 | 4.81 | 0.0002 |
| **PRA >80** | 0.25 | 0.07 | 0.92 | 0.037 |
| **N° Mismatch** |  |  |  |  |
| *5-6* | 0.37 | 0.14 | 1.03 | 0.1793 |
| *3-4* | 0.43 | 0.19 | 0.97 |  |
| *1-2* | 0.37 | 0.14 | 0.97 |  |
| *0* | 1.00 | *Reference Group* | | |

| **B) TAC+ mTORi vs TAC+MMF N=1855** | | | | |
| --- | --- | --- | --- | --- |
| **Variable** | **OR** | **95% CI** | | **p Value** |
| **Sex** |  |  |  |  |
| *F* | 0.73 | 0.54 | 0.98 | 0.038 |
| *M* | 1.00 | *Reference Group* | | |
| **Recipient age** |  |  |  |  |
| 60+ | 11.75 | 4.19 | 32.99 | <.0001 |
| *30-59* | 5.69 | 2.13 | 15.22 |  |
| *0-29* | 1.00 | *Reference Group* | | |
| **Donor age** |  |  |  |  |
| 60+ | 0.99 | 0.48 | 2.05 | 0.0013 |
| *30-59* | 0.54 | 0.27 | 1.07 |  |
| *0-29* | 1.00 | *Reference Group* | | |
| **Length of transplant hospitalization** | 0.37 | 0.24 | 0.59 | <.0001 |
| **Year of discharge** | 0.65 | 0.55 | 0.78 | <.0001 |
| **Diabetes** | 2.80 | 1.63 | 4.81 | 0.0002 |
| **PRA >80** | 0.37 | 0.14 | 0.96 | 0.0411 |
